# An Ethical Analysis of Medicalization and Infant Mental Healthcare

**DOI:** 10.1007/s11673-025-10460-5

**Published:** 2025-09-22

**Authors:** Izaak T. Lim

**Affiliations:** 1 Early in Life Mental Health Service, Monash Health, 268 Clayton Road, Clayton, VIC Australia; 2 Department of Psychiatry, School of Clinical Sciences, Monash University, 268 Clayton Road, Clayton, VIC 3150 Australia

**Keywords:** Infant mental health, Ethics, Medicalization, Infant rights

## Abstract

The field of infant mental health has been met with some scepticism by those who question the role of the mental health professions in this space. In this paper I will consider a possible ethical objection to the extension of medical jurisdiction over infancy and parenting, informed by the critical tradition of medicalization studies. In part I of the paper, I will give particular attention to three potentially harmful consequences of medicalization on infants and their families—the expansion of medical social control, the individualization of human suffering, and the pathologization of human behaviour and variation. In part II, I will provide an ethical defence of infant mental health, addressing the objections raised by a medicalization-based critique. I will conclude in part III that medicalization is not always bad for infants and their families, and that, in the case of infant mental health, there is a mutually reinforcing relationship between medicalization and infant rights claims to the fundamental conditions for pursuing a good life.

## Introduction

Some express puzzlement or even aversion to the term “infant mental health.” The idea of an “infant,” with its associations of innocence, beginnings, and hope for a better future, does not seem to fit with “mental health,” and its associations of maladjustment, stigma, and major mental illness. (Zeanah and Zeanah 2009, 5)

Infant mental health practitioners are familiar with such expressions of puzzlement and aversion. We are often asked to explain and justify what we do in a way that our colleagues working with older children, adolescents, and adults rarely are. We are faced with sceptical questions: Can babies really have mental health problems? Aren’t you really just treating the mother? How can you do therapy with babies when they can’t talk? These are reasonable questions requiring answers that both validate the questioner’s curiosity and defend the important work that we do.

One response is to cite the burgeoning research demonstrating the effects of early experience on the developing mind and the critically important role of caregiving relationships in moderating these effects. Another response is to refer to the growing evidence base endorsing interventions that help to ameliorate early emotional and relational difficulties, getting infants and their families back onto more hopeful developmental trajectories.

Yet, responses emphasizing the scientific credibility of the field can sometimes give rise to a second set of more uncomfortable questions: Aren’t you pathologizing normal behaviour? Do you medicate babies? These questions reveal a moral anxiety about the appropriate limits of medical authority and the consequences of expanding medical jurisdiction over this vulnerable population. This anxiety is not new. Its expression in the academic literature can be traced back to the late 1960 s and early 1970 s when sociologists first started mounting hostile critiques of the medical profession’s expansionist tendencies—a process they called “medicalization” (Illich 1976; Pitts 1968; Zola 1972). The concept of medicalization has been taken up by critical scholars and social movements alike and is now central to the lexicon of resistance to medical authority.

This paper is an ethical defence of infant mental health. Specifically, it is an attempt to critically evaluate the charge of medicalization as it relates to infant mental health. Its intention is not to deny that infant mental health is medicalizing of problems in infancy and parenting. Instead, by offering a nuanced appraisal of infant mental health from a moral perspective, this paper will explore the ethical ambiguity of medicalization. This effort is inspired by the work of others who have sought to situate medicalization within an explicitly bioethical discourse by asking “Can medicalization be good?” (Sadler et al. 2009) and distinguishing “good and bad forms of medicalization” (Parens 2013). Such a normative analysis has not hitherto been brought to bear on the field of infant mental health but is timely in the current context of declining trust in medical expertise.

## Part I: An Ethical Objection to the Harms of Medicalization

In this first section, I will introduce a possible ethical objection to the provision of infant mental healthcare: that the provision of such care gives rise to harms that outweigh the potential benefits of providing it. The concern about harm arises from a more general concern, that infant mental health is a form of medicalization. I will first examine the claim that the field of infant mental health is medicalizing of infancy and parenting. I will then consider the ethical significance of this claim by interpreting it in terms of harms that might occur as a result of medicalization. Specifically, I will hypothesize the harms associated with the following consequences of medicalization: 1) the expansion of medical social control, 2) the individualization of human suffering, and 3) the pathologization of human behaviour and variation.

In addressing the ethical significance of the medicalization objection, I am intentionally not seeking to answer the ontological question it poses—of whether the problems claimed by the field of infant mental health are rightly considered medical problems. This would entail a distinction between valid medicalization and invalid or over-medicalization. This distinction assumes that there is an essence to what is truly medical and non-medical and that by revealing these essential categories, we can determine the validity of medicalization (Parens 2013). In contrast, a sociological analysis of medicalization assumes a social constructionist position (Berger and Luckmann 1984), according to which no problem or event is inherently medical or non-medical but becomes so through the process of developing social agreement about the meaning of experience and nature of the world.

In this discussion I will assume a sociological perspective on medicalization and will focus on an ethical analysis of the consequences of medicalization. Specifically, I will investigate whether the medicalization of the problems claimed by the field of infant mental health can cause harm to infants and their families. However, I will not attempt to prove that these harms are certain or manifest. Instead, I will focus on demonstrating that these harms are plausible and of material significance.

## Medicalization and Related Processes

Medicalization refers to the “process by which nonmedical problems become defined and treated as medical problems, usually in terms of illness and disorders …” (Conrad 2007, 4) and is a key concept in the study of medicine as a social practice. This process applies to individual struggles and contextual stressors that become defined as the symptoms and risk factors of mental disorders and, therefore, the domain of psychiatry and related psy-disciplines (see below). The process of medicalization is closely related to the expansion of medical authority, whereby aspects of human life become the objects of medical study and expertise and thereby come under medical jurisdiction. Medicalization studies investigate the “creation, promotion, and application of medical categories (and treatments or solutions) to human problems and events …” (Conrad 2007, 13) and have revealed important examples of medical authority expanding to include jurisdiction over normal events in the human life cycle, such as pregnancy, childbirth, sex, ageing, and death.

Zola, one of the pioneers of medicalization studies, convincingly argued that medicine claims this authority (and social control) by 1) expanding what in life is deemed relevant to the good practice of medicine—for example, lifestyle determinants of health and illness, 2) retaining absolute control over certain technical procedures—for example, surgery and medication prescription, 3) retaining near absolute access to certain socially taboo areas—for example, infertility, drug addiction, and 4) expanding what in medicine is deemed relevant to the good practice of life—for example, the weight given to medical rhetoric and evidence in political debates (Zola 1976).

Related to the expansion of medical authority is the growing authority of the psy-disciplines, sometimes referred to as psychologization (De Vos 2014). The psy-disciplines are “those fields of knowledge associated with mind, mental life, and behavior” (McAvoy 2014 1527) such as psychology, psychiatry, psychoanalysis, and the psychotherapies. The pervasive influence of these psy-disciplines on Western culture and society has resulted in what has been variously described as the therapeutic ethos (Lears 1983), therapeutic culture (Illouz 2008), and triumph of the therapeutic (Rieff 1987), where all human struggles become understood through a psychological lens, recasting moral problems or “problems in living” (Szasz 1960) as psychological problems. By implication, the psychological problem requires a psychological cure, which only professionals from the psy-disciplines can provide.

For the purpose of this discussion, I will use medicalization as an umbrella term to encompass the closely related process of psychologization. While there are some clear differences, these processes have fundamental similarities in the re-definition of human problems as health problems that warrant scientific study and therapeutic intervention by an expert health professional. While it is beyond the scope of this paper to provide an historical analysis of how medicine and the psy-disciplines have enabled and influenced each other, it is plain that they have an entangled and powerful aggregate effect on how we understand human life and problems in the twenty-first century.

## Infant Mental Healthcare as the Medicalization of Infancy and Parenting

The history of the field of infant mental health reveals a tendency to expansion of scope and knowledge production that is characteristic of medicalization and is therefore worthy of some mention here. Fitzgerald and Barton (1999) have highlighted the emergence of infant mental health out of evolutionary, systems, and psychoanalytic theories into a broad and vaguely defined interdisciplinary field, which, in addition to its primary focus on the social and emotional well-being of infants and their caregivers, sees any and all of the following issues as the jurisdiction of infant mental health: “premature birth, perinatal complications, inadequate prenatal care, infant mortality and morbidity (and parental grief), physical disability, mental retardation, poverty, undernutrition, single parenting, supplemental caregiving, parental psychopathology, parental stress, abusive parenting, parental substance abuse, and marital conflict” (Fitzgerald and Barton, 1999, 21). The logic underlying this expansion of jurisdiction is that infant mental health problems tend to be multifactorial and are most likely to occur in the presence of risk factors, some of which are current and individual, and others historical and systemic. This leads to an explosion of personal information-of-interest to the infant mental health clinician, which is then subject to exploration and evaluation.

In 1994, the peak infant mental health professional body in the United States—Zero to Three—published the Diagnostic Classification of Mental Health and Developmental Disorders of Infancy and Early Childhood (DC:0-3), a counterpart to the well-known and much-maligned Diagnostic and Statistical Manual of Mental Disorders for adults (Mulrooney et al. 2019). It was the first manual for diagnosing “mental health and developmental disorders” in infancy and toddlerhood and redefined previously non-medical problems as medical problems. For example, crying and feeding difficulties became Excessive Crying Disorder and Feeding Disorder of Infancy respectively. Very young children could be grouped and labelled according to their similarities, and knowledge about these children could be gathered and organized according to these diagnostic categories.

The DC:0-3 was subsequently revised and renamed DC:0-5 in 2016 to reflect the newly extended age-range of children brought under the jurisdiction of infant mental health—that is, those from zero to five years-of-age (Zeanah et al. 2015). This renaming highlights the evolution of the term “infant,” which typically refers to the period between birth and one year, in the context of an expanding field of “infant” mental health. The DC:0-5 also extended diagnostic criteria to infants in the first year of life, and introduced several new disorders, including Overactivity Disorder of Toddlerhood and Disorder of Dysregulated Anger and Aggression of Early Childhood. These new categories represent the expansion of medical authority to claim behaviours that were previously not considered symptoms of illness, as evidence of diagnosable mental health disorders, which if properly identified can be effectively treated.

Zero to Three is ambitious in its vision for the DC:0-5, which it recommends policymakers to recognize “as the most appropriate system for diagnosis, treatment, clinical communication, and research purposes for children through 5 years old and [to] require its use in insurance plans and managed care contracts” (Zero to Three 2017, 3). In other words, it is hoped that the DC:0-5 will form the organizing basis of clinical service delivery and funding, which demonstrates the power of diagnostic classification in shaping public policy and mobilizing resources.

## The Harmful Consequences of Medicalizing Infancy and Parenting

There is a vast sociological literature that offers various critical perspectives on medicalization. While medicalization itself is a descriptive term, many prominent theorists of medicalization have taken suspicious or frankly hostile positions in relation to it, focusing their attention on the dominating and oppressive aspects of medicalization. I cannot hope to fairly represent the full range of critical scholarship here. Instead, I will focus on three major critiques that are especially pertinent to the medicalization of infancy and parenting. I will apply an ethical analysis to these sociological critiques by evaluating the ethical significance of these alleged adverse consequences of medicalization.

### The Expansion of Medical Social Control

Parents have a responsibility to provide a safe and nourishing environment for their child to grow and flourish in. Parents also have rights to raise their children according to their own values and beliefs, free from external interference. The state has a correlative duty to not interfere with how parents choose to raise their child. However, there is a limit to parental autonomy and freedom from interference, which in medical ethics is captured by the harm principle (Diekema 2004). According to this principle, the state is justified in taking control by interfering with parental decision-making authority when the child is at risk of serious harm. This harm may be direct, for example, when a parent hits or starves a child, or indirect, by failure to protect the child from harm, for example, when a parent does not take steps to limit the child’s exposure to family violence or remove dangers in the environment. The harm principle is grounded philosophically in the liberal tradition (Mill 1982 [1859]) and sets a high threshold for state interference and control.

However, the definition of harm is not stable (Epstein 1995, 371) and is determined in large part by socio-cultural beliefs and norms, which in turn are influenced by various sources of social and moral authority and knowledge about chains of causation (Dripps 1998, 9). Medicalization can have a strong influence on the definition of harm by driving the expansion of authority held by the medical profession and the production of knowledge relating to risk and causation. If something is re-defined as harmful by the medical profession, perhaps because of recent scientific attention or shift in professional consensus, the definition of harm expands. This updated definition then gets taken up by other state agencies, such as the courts and child protection services, which have the power to exercise social control directly in the lives of families, with often detrimental unintended consequences.

The medicalization of infancy and parenting illustrates this process. The rise of infant mental health has seen a growing scientific and clinical interest in the influence of the caregiving environment on infant brain development and its social-emotional correlates (Berens and Nelson 2019). There is accumulating evidence demonstrating the potentially harmful effects of adversity in the early caregiving environment (Thompson et al. 2019) and a growing anxiety around how to save infants from these environments (Lawless et al. 2014; Wilson 2002). The diagnosis of some mental disorders in infancy highlights the harm done to the infant by suboptimal caregiving, especially when the diagnosis explicitly designates a parent–child relationship problem or exposure to certain traumatic events, for example, maltreatment. While the act of diagnosis should always take into consideration the various infant-related and contextual factors that influence the development of clinical symptoms, and should never be as simple or judgemental as blaming a parent for the infant’s difficulty, recognizing caregiving behaviour as a major influence on the infant’s well-being is a central tenet of infant mental healthcare.

The increased focus on families’ risk and vulnerability from an infant mental health perspective leads to a sense of urgency around the need for other state agencies to intervene early in the infant’s life to effectively mitigate the risk associated with “pathogenic” caregiving environments. This has been described as the argument of “now or never” (Munro 2011, 69), where child protection services, for example, might be compelled to investigate parents at early signs of difficulty and swiftly intervene when infants are identified as “at-risk” of adverse events (Featherstone et al. 2014). In essence, the more ways there are to describe and classify how a child may be harmed, the broader the definition of harm becomes, which lowers the threshold for the exercise of state interference and curtailment of parental autonomy.

State interference in the family, especially the removal of children, can constitute a material harm to infants and their parents because of its disruptive effect on the continuity of the parent–child relationship, which is of primary importance to the well-being and development of the child. One of the seminal texts on this matter, *The Best Interests of the Child*—co-authored by Anna Freud, one of infant mental health’s founding mothers—provides a powerful, child-centred defence of the family’s right to parental autonomy, privacy from state surveillance and control, and the child’s entitlement to autonomous parents. The authors stress “the importance to a child of minimizing intrusions by the law on a child’s relationships to her primary caregivers—of leaving well enough alone in the service of safeguarding the continuity of psychological parent–child relationships … whether the underlying relationships are long-standing or newly formed” (Goldstein et al. 1996, xix). Furthermore, they warn that “when family integrity is broken or weakened by state intrusion, [the child’s] needs are thwarted … [and t]he effect on the child’s developmental progress is likely to be detrimental” (90). In this way, the expansion of medical social control through medicalization, and the subsequent effects on the definition of harm and basis on which the state may interfere with family integrity, threatens to cause material harm to infants and their families.

I do not argue that there are no situations in which infants should be removed from the care of their parents. While the threshold for intervention varies from jurisdiction to jurisdiction, there are certainly situations in which infants who are being harmed and are at risk of future harm ought to be removed. In such cases, the harm of removal is outweighed by the harm of failing to remove, and there is a clear ethical imperative to intervene. I do, however, argue that the definition of what constitutes harm is influenced by medicalization and the proliferation of associated scientific knowledge.

### The Individualization of Human Suffering

Medicalization brings focus to the individual person as target of assessment and intervention. In an archetypally medical example, a patient presents to a doctor with a problem of some kind, which the doctor investigates by way of collating the patient’s history, examination findings, and test results, culminating in a diagnosis of the problem and prescription of a treatment that aims to resolve the patient’s problem. However, many clinical problems, including mental health symptoms in early childhood, are the downstream products of upstream social factors (Bayer et al. 2011).

Let us consider a boy of three years who is referred to an infant mental healthcare practitioner with prolonged, destructive tantrums every day, hitting and biting other children at childcare, lack of affection for his parents, and inappropriately affectionate behaviour with adult strangers (the problem). The immediate context of these problems is his return to the care of his parents six months ago after a period of eighteen months in multiple foster care placements, on a background of removal from the family home at twelve months of age due to his parents’ heavy drug use, which had resulted in significant physical and emotional neglect (individual risk factors). Importantly, both parents have their own histories of being abused and neglected as young children, growing up in dangerous and under-resourced neighbourhoods, leaving school early, and experiencing chronic unemployment and financial hardship (social risk factors).

Let us imagine that the clinician provides a combination of parenting skills sessions with the boy’s parents and trauma-informed play sessions with the boy and his parents that focus on rebuilding a safe and trusting caregiving relationship. Over the course of twelve months of weekly sessions, the relationship between the boy and his parents improves, as does his aggression. Ostensibly, the treatment has been a success, and yet, it has not done anything to ameliorate the upstream factors that led to this boy’s problems in the first place—poverty, socioeconomic inequality, family violence, poor school retention, and unequal access to childcare and other family support services.

This example demonstrates how medicalization can decontextualize clinical problems and thus “individualizes what might otherwise be seen as collective social problems” (Conrad 1992, 224). Of course, it would be unrealistic to expect the clinician to attend to all social risk factors relating to the presenting problem, though they may be aware of them. Commonly, social risk factors are considered in the process of clinical assessment and formulation by way of attempting to understand the nature and origins of the presenting problem. This provides an intellectual bridge between the upstream and the downstream, yet the clinical encounter is grounded in the immediacies of individual experience, and most infant mental health practitioners are trained to intervene at the level of the child and their family. Indeed, it would be bamboozling for a family who presents with a symptomatic infant to be met by a public health advocate with a campaign for extending parental leave or free and universal childcare.

How, then, might material harm be caused to infants and their families by individualizing human suffering, when clinical treatments may be effective in relieving the suffering of the individual patient? Here, the fair distribution of resources, another central pillar of medical ethics, comes to the fore (Beauchamp and Childress 2013).

There is only a finite quantity of public resources that can be spent on social programmes. In the year 2018–2019, prior to the monumental impact of the COVID-19 pandemic, health expenditure accounted for approximately 16.1 per cent of all Australian government expenditure (Australian Institute of Health and Welfare 2020), which highlights the significant expense of providing healthcare and the burden this has on the economy as a whole. While the delivery of healthcare is obviously important, the higher the costs of healthcare, the less resources there are to fund programmes that address the social determinants of health, including education, housing, family services, and employment initiatives. Therefore, the resources required to deliver infant mental healthcare are resources that cannot be put towards programmes that may prevent the clinical problems seen by infant mental healthcare services. In this way, harm can be done by failing to prevent the suffering of other and future infants and families. As elegantly distilled by Mills (2015):

[A] grave danger in the medicalization and psychiatrization of poverty is the confusion of cause and effect—for while poverty may lead to psychological distress, correcting only the psychological issues will not necessarily correct the conditions of poverty that lead to the distress in the first place or the political-economic forces that produce and sustain poverty and economic marginalization. Thus, not only does such psychiatrization overlook the conditions of inequality and poverty that may lead to distress initially, it may actually allow such conditions to persist or worsen. (218)

### The Pathologization of Human Behaviour and Variation

Conrad, who has been prolific in his contribution to medicalization studies, has drawn attention to the transformation of human differences into pathologies as an important consequence of medicalization:

Differences in learning styles become learning disabilities or ADHD; divergences in sexual desires or performance become sexual dysfunctions; extremes of behavior become sexual, shopping, or Internet addictions … Virtually any human difference is susceptible to being considered a form of pathology, a diagnosable disorder, and subject to medical intervention” (Conrad 2007, 148).

This is evident in the growth of diagnostic categories in the DC:0-5, which recasts unsettledness in a newborn, restlessness in a toddler, and shyness in a preschooler as possible symptoms of mental disorder, if associated with distress and functional impairment experienced by the child or family. The great danger, Conrad warns, is that medicalization “diminishes our tolerance for and appreciation of the diversity of human life” (Conrad 2007, 148). In other words, social norms around acceptable ways to think, feel, and behave are narrowed by medicalization, and the threshold for needing professional help with ordinary problems in living is lowered.

This is reminiscent of an argument put by Illich, one of the earliest and most polemic critics of medicalization, who warned that the capacity of individuals and societies to care for themselves and cope with sickness, pain, and death would be undermined by medicalization (Illich 1975). In his view, dependence on medical care leads to “social iatrogenesis,” the process by which “medical practice sponsors sickness by reinforcing a morbid society that encourages people to become consumers of curative, preventive, industrial and environmental medicine” (Illich 1976, 33). The growing number of functional somatic syndromes, such as chronic fatigue syndrome, fibromyalgia, and irritable bowel syndrome, which present with persistent and debilitating physical symptoms without an identifiable organic cause, could be compelling realizations of Illich’s prediction. According to psychiatrists Barsky and Borus (1995), medicalization fosters somatization by amplifying mild and benign bodily distress, alarming us about the potential pathological significance of minor physical symptoms and uncomfortable body states, leading to the enthusiastic pursuit of medical scrutiny and adoption of medical intervention.

Along similar lines, the pathologization of infant and parenting behaviours may reduce the capacity—actual and self-perceived—of families to overcome difficulties without professional help. The emergence of sleep and settling consultants and sleep schools, for example, exemplify the growing domination of professional services in a space that has traditionally been managed within families. When a family encounters challenges with an infant’s sleep routine, if they believe there is a serious problem at hand requiring urgent professional attention, such as a residential stay at a specialist infant sleep facility, they may be less likely to endure some struggle at home, attempt solutions of their own devising, and thus build their sense of competence by working through the challenge independently. In this way, medicalization can adversely influence parents’ sense of themselves as parents who can manage challenges in child-rearing competently and independently. This may result in fewer experiences of actually overcoming difficulties, where parents can develop mastery of parenting skills through persistence and learning from failure. This is what Illich referred to as the “disabling” effect of the professions (Illich 1977).

Another important potential harm to consider is the impact of pathologization on the child’s developing sense of self. The stigma associated with mental health diagnosis and treatment is well studied in adult populations (Sickel et al. 2014). Self-stigma—the internalization of stigma by the person to whom the prejudice and discrimination is directed—has detrimental effects on that person’s self-esteem and self-efficacy—a sense of “why try?” (Corrigan et al. 2006). The endorsement of stereotypes by the stigmatized person can also lead to self-discrimination, resulting in self-isolation, reduced healthcare service use, poorer health outcomes and poorer quality of life (Corrigan and Rao 2012). The impact of self-stigma on children is less well understood, although there is some research to show that psychiatric diagnosis can have a significant influence on young persons’ self-concept and social identity (O’Connor et al. 2018). While some beneficial effects of diagnosis have been identified, the risks of self-threat and self-devaluation, and social alienation, invalidation and stigmatization have equally been noted, leading the authors to urge caution and conclude that “[i]n deciding whether to pursue or accept a diagnosis, clinicians, parents and young people themselves must trade off its potential risks and benefits” (O’Connor et al. 2018, 107).

For infants who come under medical jurisdiction for mental health diagnosis and treatment, similar caution should be exercised, as there is a risk that this experience may cause the infant to internalize a sense of badness, wrongness, or damage at an even earlier stage of their self-development. Instead of being celebrated for their differences, or even accepted despite them, these infants may receive an unspoken message about needing to be fixed, and grow up thinking—“Am I so broken that I needed therapy even as a baby?” or “Am I so unlovable that my parents needed help to learn how to love me?” In this way, medicalization, by way of pathologizing human behaviour and variation, may cause harm to infants and families by undermining parents’ sense of competence and infants’ developing sense of self.

Of course, there are multiple influences on the infant’s developing sense of self, and it is widely accepted in the developmental sciences that one of the strongest influences is the infant’s direct experience with caregivers (Hobson 2004; Stern 2000). Infants who experience hostility or frank maltreatment in the context of the caregiving relationship, for example, may internalize beliefs about being defective or unlovable that go on to affect future relational functioning, risk of psychopathology, and a range of other health and social outcomes (Cohen et al. 2016). So, while the risk of harm associated with pathologization is certainly plausible, it is also true that risk to the infant’s developing sense of self may be the reason for, rather than the result of, mental health treatment.

## Part II: An Ethical Defence of Medicalization and Infant Mental Healthcare

Having hypothesized the potential harms of medicalization, in this second section I will first critically evaluate the ethical concerns raised in part I and thus seek to offer a nuanced and balanced analysis of medicalization as it relates to infant mental healthcare by highlighting its complexity and ethical ambiguity.

### Response to the Potential Harms of Medicalization

#### The Expansion of Medical Social Control

In the previous section, I explored the specific example of child protection services using infant mental health concepts to justify intervening in families as an illustration of how medicalization could be used to exercise social control. While this is a reasonable concern, it does not necessarily follow that medicalization is wholly bad. Some degree of social control is necessary for an organized and cohesive society, and managing undesirable human problems is an important aspect of this. Medicalization is but one approach to managing human problems, and as I will argue below, it is often preferable to other approaches. First, however, I will highlight the need for a complex, rather than polemical, analysis of medical authority.

It is an oversimplification to characterize medicalization as a unidimensional, top-down process imposed by doctors onto passive or unwilling patients (Ballard and Elston 2005). Sophisticated sociological research has revealed that patients, including children and their supporters, can be enthusiastic agents of and advocates for medicalization (Beeker et al. 2020; Bell 2017). Notable examples of where patients have led social movements to have their problems recognized as medical conditions, sometimes in the face of resistance from the medical profession itself, include Alzheimer’s disease (Fox 1989), chronic fatigue syndrome (Broom and Woodward 1996), and post-traumatic stress disorder (Scott 1990). As observed by Zola (1976), medicalization “is as much a result of medicine’s potential as it is of society’s wish for medicine to use that potential” (213).

Broom and Woodward (1996) make much of the distinction between medicalization, which they do not see as necessarily harmful, and medical dominance, which they see as more problematic. Medical dominance, by their account, is evident “when the doctor’s priorities and views dictate what transpires” (361) in the clinical consultation and “when the opinions and interests of doctors determine policy decisions” (361). They cite examples of where doctors may approach non-medicalized issues in a paternalistic way, i.e., doctor-knows-best, and examples of where doctors approach medicalized issues in a collaborative and constructive way. Their research elucidates the potential of medicalization to enhance patients’ sense of coherence and legitimacy in relation to their experience of symptoms, concluding that “the challenge for both doctors and patients is to mobilize medicalisation when it is appropriate, and to do so in a collaborative manner rather than under the sway of medical dominance” (376).

In this way, while medicalization expands the scope for exercise of medical authority, with obvious effects on social norms, the more salient ethical issue is the way this authority is exercised. This sentiment is echoed by some feminist scholars of medicalization who, while suspicious of the way medical culture and institutions often control women and deflect attention from unjust social conditions, also acknowledge that medicalization and healthcare have the potential to empower women when women’s needs and preferences are central to their design and delivery, e.g., Riessman (1983), Purdy (2001), Morgan (1998), Treichler (1990), and Sawicki (1991).

It may well be that in creating expertise around infancy and parenting, there is some giving over of epistemic authority to infant mental health professionals, which gives legitimacy to a more controlling form of authority over families. However, I would argue that neither form of authority is unilaterally imposed onto families, and that the culture and institutions of infant mental health are, by and large, respectful of the needs and preferences of infants and their parents. For the most part, families opt-in to infant mental health services on a voluntary basis, and engaging the family in a mutually respectful and trusting therapeutic relationship is vital to any successful treatment (Horvath 2000). By necessity, infant mental health professionals must be sensitive to parents’ need for control and sense of self-efficacy or they risk the family’s disengagement from treatment altogether. In other words, parents can choose to opt-in or opt-out of infant mental healthcare, and the overt exercise of medical dominance is likely to push parents into opting-out and is therefore self-defeating.

In essence, families who voluntarily choose to seek the help of infant mental health professionals are not ceding autonomy by doing so, they are exercising it. Even if this means giving over some epistemic authority to a professional, if done with the agreement of a parent who is free to terminate the therapeutic engagement at any time of their choosing, I submit that no autonomy is lost. On the contrary, it would be an exercise of medical dominance for the health professional to withhold the benefits of treatment because of their own moral anxiety about exercising medical authority and social control (Broom and Woodward 1996).

#### The Individualization of Human Suffering

In the previous section, I explained how a focus on delivering clinical services to individuals with symptoms can steal attention from the upstream social factors that made a causal contribution to these symptoms in the first place. While this is a reasonable concern, it does not necessarily follow that we should not deliver clinical services to those with mental health difficulties. Instead, we are left with an ethical dilemma around fair resource allocation.

On one hand, there is an obligation to alleviate current suffering. On the other hand, there is an obligation to prevent future suffering. The question, as elegantly put by Radden (2018) is

… [w]ith a finite set of resources, what proportion should be directed toward the goal of eliminating disorders entirely through efforts at early (primary) prevention, and what toward relieving the symptoms and societal costs of those already suffering from the disorder? (129)

Public health psychiatrists have argued that the only sustainable method for reducing the burden of mental health problems is prevention, especially in light of the evidence supporting the effectiveness of social policy interventions to improve nutrition, housing, education, and economic insecurity in reducing the incidence of mental and behavioural disorders (Saxena et al. 2006). Furthermore, there appears to be a growing reliance on notions of evidence-based policy and cost-effectiveness calculations to resolve the question of how to allocate resources.

This essentially utilitarian perspective fails to address the question of how clinical health professionals should discharge their paramount duty—to provide care to the patients before them—without sufficient resources. It is beyond the scope of this paper to resolve this question. However, I would argue that while clinical health professionals do have concurrent obligations to those currently suffering and those who might suffer in future, their overriding duty is to the former, which is in keeping with a conventional, duty-based clinical ethics, as reflected in various ethical codes and guidelines, e.g., American Medical Association (2016, 2), Medical Board of Australia (2020, 6). While clinicians should be responsible custodians of healthcare resources, there is a prima facie duty to provide treatment to those in need. Furthermore, as I will elaborate below, the obligation to provide treatment is not always in conflict with the obligation to prevent future suffering, as attending to the former can support the latter.

Zola (1976) noted in his early conception of medicalization that medical knowledge is given particular weight in political debates, which has the potential to shape policy decisions that influence upstream social determinants of mental health. For example, clinical research on the effect of child abuse and neglect, e.g., Humphreys, King, and Gotlib (2019), family violence, e.g., Schechter, Willheim, Suardi, and Serpa (2019), and poverty, e.g., Piccolo and Noble (2019), on infant development and future mental health outcomes can help galvanize public support and political will to strengthen policy in these areas (Knitzer 2007). Organizations such as Zero to Three in the United States are especially active in this kind of advocacy to policymakers, drawing on scientific knowledge to develop policy recommendations that advance the interests of infants and their families (Zero to Three, n.d.). In this way, the body of medical knowledge that comes from treating individuals who suffer the downstream effects of social problems can be used in political advocacy and policy development to help ameliorate those social problems.

Finally (and of particular relevance to clinical infant mental health practice), providing treatment to infants and families with current difficulties has both immediate and future benefits and is thus essentially preventive (Fonagy 1998). The primary purpose of early intervention with young children and their families is to reduce the future symptom burden by acting early in the course of social and emotional development, while symptoms are still mild or even subclinical (Little 1999). There is also an intergenerational dividend to infant mental health interventions in that intervening to improve the quality of the parent–infant relationship will leave the infant with a better relational model to bring to the parenting role when they have children of their own (Narayan et al. 2021; van Ijzendoorn et al. 1995). In this way, the intergenerational transmission of relational difficulties can be interrupted, and future suffering averted.

#### The Pathologization of Human Behaviour and Variation

In the previous section, I explored how the pathologization of difficulties in infancy and parenthood could lead to a reduction in self-efficacy, a dependence on professionals, and the internalization of stigma. While this is a plausible concern, it does not necessarily follow that infancy and parenthood should be completely demedicalized. In this section I will elaborate the distinction between pathologization and medicalization and argue that, while certain harms may come with pathologization, to dispense with medicalization altogether would risk throwing the baby out with the bathwater.

Sholl (2017) distinguishes between pathologization and medicalization, defining pathologization as “the ways in which certain conditions come to be labelled as pathological by medical institutions (definitions), in the clinic (diagnoses), or by self-labelling” (268) and medicalization as the “various types of medical responses and interventions or treatments that are justified in relation to health concerns” (268-269). This conception is reminiscent of Sadler et al.’s (2009) test for establishing claims of medicalization, which centres on the prominence or importance of medical justifications for a given behaviour or practice, for example, exercising to stay healthy, reduce blood pressure, or keep one’s weight down (Sadler et al. 2009). Sholl’s medicalization is what Conrad might refer to as “healthicization,” which the latter distinguishes from medicalization by emphasizing the redefinition of behaviours and lifestyles in relation to medical events (healthicization), as compared to the redefinition of social or natural events into medical problems (medicalization) (Conrad 1992).

On Sholl’s account, medicalization is a much broader set of practices than pathologization, and he offers several examples of where medicalization occurs without pathologization and vice versa. Obvious examples of situations where medical intervention is well-accepted and considered useful in the absence of pathology include pregnancy, baldness, and vaccination. Of particular relevance to infant mental healthcare, however, is the provision of treatment to those “at risk” of developing a disease or disorder, referred to as primary prevention. As explained above, infant mental health interventions are preventive by nature, even when focused on treating current problems, by virtue of the very early life stage at which the intervention occurs and the intergenerational character of relational difficulties. Infants do not need to suffer diagnosable psychopathology to benefit from mental health intervention, so not all infants receiving mental health intervention have psychopathology. Therefore, infant mental health intervention does not necessarily imply pathology.

That said, a key aspect of the medicalization of infancy and parenting as I have described it is the growth of psychiatric diagnostic labels applicable to very young children, which is more clearly pathologizing. While a detailed analysis of the validity, reliability, and utility of psychiatric diagnosis in infancy is beyond the scope of this paper, I concede that this facet of medicalization is fairly characterized as one of its more troubling and should be subject to rigorous examination. Indeed, the usefulness and appropriateness of psychiatric diagnosis in infancy has been the subject of significant debate within the infant mental health profession, even at the highest levels of peak international organizations (von Klitzing 2017; Zeanah et al. 2017).

Of course, it is not just in infancy that the value of psychiatric diagnosis is contested; the importance and shortcomings of any and all psychiatric classification systems have been well-explored in the academic literature, e.g., Insel et al. (2010), Kendell (1975), Maj (2011), Mathis (1992), Wykes and Callard (2010). Typically, defenders of diagnosis cite its practical value in how psychiatrists communicate with patients and each other and its potential to reassure patients that their situation is not unique or inexplicable, reduce inappropriate feelings of blame, enable people with similar problems to connect, and stimulate service improvement and innovation, among other benefits (Craddock and Mynors-Wallis 2014).

Proponents of psychiatric diagnosis in infancy and early childhood argue that developmentally sensitive diagnoses can help include young children in the theory and practice of mental health, thereby facilitating access to much needed clinical services and participation in important research studies (von Klitzing 2017). There is also some attention given to the risk of not labelling a clinical syndrome, in particular, the alternative labels that may be given to children with behavioural or emotional difficulties, such as “bad,” “weird,” “manipulative,” or “spoiled” (Zeanah et al. 2017). In other words, in the absence of a “scientific” label, an adverse moral one will be applied.

It may therefore be more detrimental to the child’s developing sense of self to *withhold* a psychiatric diagnosis, especially when introducing clinical concepts could help to displace parental attributions of blame, naughtiness, or malevolence to the child. Indeed, there is empirical evidence to suggest that treatment engagement is higher when parents know their child’s diagnosis (Peters et al. 2005). If these purported benefits of psychiatric diagnosis do not flip the stigma argument in favour of making a diagnosis, they should at least give pause to those who would dispense with diagnostic classification altogether, e.g., Timimi (2014). While the pathologization aspect of infant mental healthcare is of concern and requires the careful attention of clinicians who are involved in making diagnoses in young children and explaining these to their families, it does not outweigh the good that may come from medicalization as a whole.

## Part III: The Benefits of Medicalization, Infant Rights, and the Role of Personal Values

I have argued that while medicalization has its ethical shortcomings, it is far too complex and multifaceted a phenomenon to be wholly bad. I have also argued that compared to other prominent models for understanding and managing human problems, medicalization is preferable when it comes to infant mental health difficulties. In this final section, I will lay out some of the ethical benefits associated with the medicalization of infancy and parenting, and synthesize these with a rights-based argument for the provision of infant mental healthcare.

In an important early contribution to medical sociology, Parsons (1951) developed the concept of the sick role—a set of socially negotiated norms and expectations associated with the experience of illness. These norms include certain social protections and allowances granted to people who are assigned to this role. For example, the sick person is exempted from responsibility for her own state, which is in sharp contrast to the moral model. Using Parson’s formulation, assigning the sick role to infants and families with emotional and relational difficulties reduces individual blame for these difficulties (i.e., caused by “flawed character” or “bad parenting”). In the clinical encounter, this translates to the clinician adopting a non-judgemental stance, which emphasizes curiosity and acceptance over criticism and punishment. Empirically, these “common factors” of the therapeutic approach is more likely to lead to positive change for the person in the sick role and is therefore ethically underpinned by principles of care and beneficence (Wampold 2015).

Assignment of the sick role also signifies the need for help (Parsons 1951) and thus demands that the sick person be taken seriously by healthcare professionals and society. In this way, the use of psychiatric diagnosis as a means of denoting sickness has the potential to recast “the whingeing baby,” “the naughty toddler,” and “the overly shy child”—who might otherwise be condemned or dismissed—into troubled children who are entitled a professional service. There are serious moral implications to recognizing this need for help, as it confers obligations on others to provide it, or at least facilitate access to it, and legitimates claims made by the sick person to receive it (i.e., moral rights, see below).

Becoming a client of a professional service reinforces the seriousness of the moral relationship between the help-seeker and the help-giver by invoking a set of professional duties on the help-giver, for example, duties of confidentiality, truth-telling, and non-exploitation. In short, assigning the sick role to infants and families with emotional and relational difficulties creates a set of moral relationships between the infant and society, such that there is an ethical imperative to help the infant (i.e., the duty to relieve or prevent suffering and the duty to protect the vulnerable) and such that a moral wrong has occurred if this help is withheld (i.e., violation of a sick person’s right to receive care and possible harm by act of omission).

Moreover, the sick role implies the possibility of and intention to change (Parsons 1951). This may have empowering effects on families who have felt hopeless and helpless in the face of their suffering. Notions of treatment and recovery invite the family to imagine themselves in a state of less distress and impairment, with an eventual return to well-being and flourishing, or at least more normal functioning. Importantly, seeking treatment is a way of exercising one’s agency and reclaiming mastery of and control over one’s situation. In this way, infant mental healthcare is an intrinsically optimistic endeavour, which, at its best, works compassionately and collaboratively with families to give their infants the best possible start in life.

Konnoth, a legal scholar, has recently written about “medical civil rights” as a powerful means of extracting the emancipatory potential of medicalization (Konnoth 2020). He argues that the increasing use of medical concepts to seek rights and benefits should be a welcome phenomenon, contrary to the view taken by some other legal scholars who emphasize the harms and ignore the benefits of medicalization. He cites three key arguments in defence of his position: a) legal protections that accompany medical status are more robust than those received by other vulnerable groups, such as the poor, the unemployed, or racial minorities, b) society holds the medically disadvantaged relatively blameless for any disadvantage, and c) medical language creates a sense of objectivity and legitimacy for those invoking it. He concludes that the synergy of medical recognition and legal rights-giving can “yield powerful formal rights mechanisms and reinforce normative views about who or what should be blamed for medical harm—in most cases, fate and society” (Konnoth 2020, 1263).

While Konnoth explicitly refers to legal rights, his arguments could equally apply to the moral rights of medicalized infants. The medical recognition of emotional and relational difficulties in infancy brings with it a moral seriousness and sense of urgency to the provision of help to infants and families with these difficulties. For example, the field of infant mental health has made a significant contribution to public awareness of the developmentally specific needs of infants and the critical importance of the early years on subsequent health and well-being, e.g., Leach (2017). Furthermore, the accumulating scientific evidence on the environmental conditions required for optimal social and emotional development strengthens policy advocacy for infants and their families. In other words, empirical medical knowledge and practice helps to legitimate normative infant rights claims to the fundamental conditions for human flourishing.

I have outlined above some of the benefits of medicalization and have attempted to sketch out a mutually reinforcing relationship between medicalization and infant rights. However, I have not accounted for families who do not wish to live a medicalized life. In this final section, I will offer some preliminary thoughts on this conundrum in the knowledge that I cannot hope to resolve it fully here.

In most cases, the desirability of medicalization is a judgement that cannot be made without recourse to personal values. Individuals come to their own conclusions about whether they wish to pursue medicalized solutions to the problems they experience. For example, if a person feels sad and anxious every day, they may choose to seek the assistance of a doctor. Equally, this person may value their stoic endurance above all else and choose to accept their mental suffering without any assistance from others. These divergent choices arising from differences in personal values are both reasonable and illustrate that judgements of this sort should usually be made by the person experiencing the problem.

Clearly this cannot be the case for infants, who, even if they have preferences, cannot be said to have values per se, let alone the capacity to make rational judgements and act on them. Choices affecting the life of the infant are entrusted to her parents. Yet, when an infant has a serious health problem, it seems obvious that the assistance of a health professional should always be sought, the personal values and preferences of parents notwithstanding. This brings us to the problem of where lie the limits of parental discretion in decision-making on the child’s behalf.

Consider the following comparison. It would be uncontroversial for a parent to pursue medical diagnosis and treatment if their infant had a serious physical health problem. If an infant is in pain due to a healing bone fracture, for example, it would seem reasonable for her parents to request analgesic medications from a doctor. However, it *would* be controversial for parents to choose non-medical approaches where a well-established medical approach exists. In the example of the infant who is in pain due to a healing bone fracture, it would be unreasonable for her parents to refuse analgesic medications from a doctor on the basis that they believe she should learn to endure physical pain. Indeed, withholding appropriate medical care from an infant would be considered sufficient cause in many jurisdictions for the state to intervene through child protection services. Similarly, if an infant has emotional symptoms due to psychological trauma, it would seem reasonable for her parents to request psychotherapy from an infant mental health professional, and unreasonable to refuse it on the basis that they believe she should learn to endure psychological pain.

While physical suffering is still sometimes (mis)taken to be more serious that emotional suffering, I would argue that there is no ethically relevant difference between a parent’s response to their child’s physical pain and their response to emotional pain. Both forms of suffering compel some action on the parents’ part, and seeking the assistance of a healthcare professional is the most appropriate course. This leads us to conclude that while adults may exercise choice around whether they opt-in or opt-out of medicalized approaches to their own emotional suffering, parents cannot exercise this choice as freely when it comes to responding to their infant’s emotional suffering. To withhold professional help would constitute a violation of the infant’s right to receive appropriate healthcare and may cause harm by act of omission (i.e., poorer developmental outcomes or worsening emotional and relational symptoms).

Determining exactly how much choice parents have in relation to the kinds of suffering infants may experience is beyond the scope of this paper. However, an important implication of recognizing the moral equivalence of physical and emotional harm is the state’s obligation to treat parental failures to protect infants’ emotional well-being with the same seriousness as failures to protect their physical well-being. In the same way that parental refusal to access medical care when an infant is showing signs of physical disorder might precipitate a state response through child protection services, the medicalization of infant emotional and relational difficulties imposes an obligation on the state to respond when there is parental refusal to access medical care when an infant is showing signs of mental disorder.

An objection to this argument might be that signs of mental disorder in infancy are harder for parents to recognize than signs of physical disorder, and it is therefore unfair to hold parents to the same level of responsibility when it comes to facilitating the infant’s access to healthcare. There is merit to this concern, as there is still relatively poor awareness of infant mental health difficulties and services. Yet, in principle, if a parent has been advised on the infant’s need for mental healthcare by an informed professional, such as a family doctor, home visitor, or early childhood educator, they have an obligation to facilitate the infant’s access to help or at least take reasonable steps to do so.

A further implication of medicalization relates to the allocation of resources towards clinical infant mental health services and public health initiatives aimed at population-level risk factors for infant mental health problems. As discussed in part I of this paper, resource limitations necessitate difficult decisions about how to maximize benefit for the greatest number of people. The medicalization of infancy and parenting dovetails with economic and child development research that emphasizes the long-term value of optimizing the early childhood environment (e.g., Knudsen et al. 2006; Strong Foundations Collaboration 2019). Integrated policy programmes that invest in various domains of the early childhood system, such as access to medical care, early childhood care and education, family support and parenting education, and mental health and social-emotional development services, have the potential to yield dividends across the lifespan and intergenerationally in multiple domains (e.g., mental and physical health, educational outcomes, economic participation) (Nagle 2019). The medical model elevates the seriousness of emotional and relational difficulties in infancy by endowing language and conceptual infrastructure that can stimulate clinical research programmes, public policy interest, and government investment, as has become increasingly evident especially in high-income countries (e.g., Daníelsdóttir and Ingudóttir 2022; Department of Health and Social Care 2024; Hogg and Moody 2023; The Royal Australian and New Zealand College of Psychiatrists 2023).

## Conclusion

The image of an infant on a psychiatrist’s couch seems silly, and the juxtaposition of mental illness and babyhood makes us feel uncomfortable. On further reflection, however, providing help to troubled families and trying to get infants off to a reasonable start are not such bad ideas after all (Zeanah, 1993, ix).

There is a strong ethical warrant for the provision of infant mental healthcare. Infant mental health difficulties threaten the fundamental conditions for human flourishing. Based on their very strong interest in human flourishing, infants have a legitimate rights-based claim to mental healthcare. The infant’s right to mental healthcare imposes a moral duty on parents and healthcare professionals to ensure the infant has access to it when needed.

I conclude that this ethical warrant withstands the charge of medicalization. Medicalization is a complex and ethically ambiguous phenomenon that brings both potential harms and benefits to infants and their families. The harms do not clearly outweigh the benefits of medicalizing problems in infancy and parenting, and it is possible to mitigate some of medicalization’s more problematic aspects without losing its benevolent, care-oriented aspects.

I conclude that there is a prima facie case for defaulting to an infant mental health approach in response to infants with emotional and relational difficulties and a mutually reinforcing relationship between the medicalization of emotional and relational difficulties in infancy and the infant’s rights claim to mental healthcare. This rights-based framework and ethical appraisal of medicalization converge in favour of providing infant mental healthcare.

## Data Availability

Not applicable as no data was collected for this research.

## References

[CR1] American Medical Association. 2016. *Code of medical ethics opinions on patient-phy**sician r**elationships*. https://www.ama-assn.org/system/files/2019-01/code-of-medical-ethics-chapter-1_0.pdf. Accessed 15 Sept 2025.

[CR2] Australian Institute of Health and Welfare. 2020. *Health expenditure Australia 2018–19.* Health and welfare expenditure series no. 66. https://www.aihw.gov.au/getmedia/a5cfb53c-a22f-407b-8c6f-3820544cb900/aihw-hwe-80.pdf.aspx?inline=true. Accessed 15 Sept 2025.

[CR3] Ballard, K., and M. Elston. 2005. Medicalisation: A multi-dimensional concept. *Social Theory & Health* 3: 228–241.

[CR4] Barsky, A., and J. Borus. 1995. Somatization and medicalization in the era of managed care. *Journal of the American Medical Association* 274: 1931–1934. 8568987

[CR5] Bayer, J., O. Ukomunne, N. Lucas, M. Wake, K. Scalzo, and J. Nicholson. 2011. Risk factors for childhood mental health symptoms: National longitudinal study of Australian children. *Pediatrics* 128(4): e865–879. 21890824 10.1542/peds.2011-0491

[CR6] Beauchamp, T., and J. Childress. 2013. *Principles of biomedical ethics*, 7th ed.). Oxford University Press.

[CR7] Beeker, T., A. Witeska-Młynarczyk, S. te Meerman, and C. Mills. 2020. Psychiatrization of, with and by children: Drawing a complex picture. *Global Studies of Childhood* 10(1): 12–25.

[CR8] Bell, A. 2017. The gas that fuels the engine: Individuals’ motivations for medicalisation. *Sociology of Health & Illness* 39(8): 1480–1495. 28815633 10.1111/1467-9566.12607

[CR9] Berens, A., and C. Nelson. 2019. Neurobiology of fetal and infant development: Implications for infant mental health. In *Handbook of infant mental health*, 4th ed. Edited by C. Zeanah. The Guildford Press.

[CR10] Berger, P., and T. Luckmann. 1984. *The social construction of reality: A treatise in the sociology of knowledge*. Penguin.

[CR11] Broom, D., and R. Woodward. 1996. Medicalisation reconsidered: Toward a collaborative approach to care. *Sociology of Health & Illness* 18(3): 357–378.

[CR12] Cohen, L., O. Leibu, T. Tanis, F. Ardalan, and I. Galynker. 2016. Disturbed self concept mediates the relationship between childhood maltreatment and adult personality pathology. *Comprehensive Psychiatry* 68: 186–192. 27234201 10.1016/j.comppsych.2016.04.020

[CR13] Conrad, P. 1992. Medicalization and social control. *Annual Review of Sociology* 18: 209–232.

[CR14] Conrad, P. 2007. *The medicalization of society: On the transformation of human conditions into treatable disorders*. Johns Hopkins University Press.

[CR15] Corrigan, P., and D. Rao. 2012. On the self-stigma of mental illness: Stages, disclosure, and strategies for change. *Canadian Journal of Psychiatry* 57(8): 464–469. 22854028 10.1177/070674371205700804 PMC3610943

[CR16] Corrigan, P., A. Watson, and L. Barr. 2006. The self-stigma of mental illness: Implications for self-esteem and self-efficacy. *Journal of Social and Clinical Psychology* 25(8): 875–884.

[CR17] Craddock, N., and L. Mynors-Wallis. 2014. Psychiatric diagnosis: Impersonal, imperfect and important. *British Journal of Psychiatry,* 204(2): 93–95. 10.1192/bjp.bp.113.133090 24493652

[CR18] Daníelsdóttir, S., and J. Ingudóttir. 2022. *The first 1000 days in the Nordic countries: Policy recommendations*. Nordic Council of Ministers.

[CR19] Department of Health and Social Care. (U.K.) 2024. *Improving the mental health of babies, children and young people: A framework of modifiable factors to guide promotion of good mental health in babies, children and young people*. DHSC. https://www.gov.uk/government/publications/improving-the-mental-health-of-babies-children-and-young-people. Accessed 15 Sept 2025.

[CR20] De Vos, J. 2014. Psychologization. *In Encyclopedia of Critical Psychology*, edited by T. Teo, 1547–1551. New York: Springer.

[CR21] Diekema, D. 2004. Parental refusals of medical treatment: The harm principle as threshold for state intervention. *Theoretical Medicine and Bioethics* 25: 243–264. 15637945 10.1007/s11017-004-3146-6

[CR22] Dripps, D. 1998. The liberal critique of the harm principle. *Criminal Justice Ethics* 17(2): 3–1.

[CR23] Epstein, R. 1995. The harm principle—and how it grew. *The University of Toronto Law Journal* 45(4): 369–417.

[CR24] Featherstone, B., J. Morris, and S. White. 2014. A marriage made in hell: Early intervention meets child protection. *British Journal of Social Work* 44: 1735–1749.

[CR25] Fitzgerald, H., and L. Barton. 1999. Infant mental health: Origins and emergence of an interdisciplinary field. In *WAIMH handbook of infant mental health: Volume 1, Perspectives on infant mental health*, 1^st^ ed. Edited by J. Osofsky and H. Fitzgerald. New York: John Wiley & Sons.

[CR26] Fonagy, P. 1998. Prevention, the appropriate target of infant psychotherapy. *Infant Mental Health Journal* 19(2): 124–150.

[CR27] Fox, P. 1989. From senility to Alzheimer’s Disease: The rise of the Alzheimer’s Disease movement. *The Milbank Quarterly* 67(1): 58–102. 2682166

[CR28] Goldstein, J., A. Solnit, S. Goldstein, and A. Freud. 1996. *The best interests of the child*. New York: Free Press.

[CR29] Hobson, P. 2004. *The cradle of thought: Exploring the origins of thinking*. Oxford University Press.

[CR30] Hogg, S., and J. Moody. 2023. *Understanding and supporting mental health in infancy and early childhood—a toolkit to support local action in the UK*. London: UNICEF UK.

[CR31] Horvath, A. 2000. The therapeutic relationship: From transference to alliance. *Journal of Clinical Psychology* 4(4): 163–173. 10.1002/(sici)1097-4679(200002)56:2<163::aid-jclp3>3.0.co;2-d 10718600

[CR32] Humphreys, K., L. King, and I. Gotlib. 2019. Neglect. In *Handbook of infant mental health*, 4th ed., edited by C. Zeanah. Guilford Press.

[CR33] Illich, I. 1975. The medicalization of life. *Journal of Medical Ethics* 1: 73–77. 809583 10.1136/jme.1.2.73 PMC1154458

[CR34] ____. 1976. *Medical nemesis: The expropriation of health*. Random House.

[CR35] ____. 1977. Disabling professions: Notes for a lecture. *Contemporary Crises* 1: 359–370.

[CR36] Illouz, E. 2008. *Saving the modern soul: Therapy, emotions, and the culture of self-help*. University of California Press.

[CR37] Insel, T., B. Cuthbert, M. Farvey, R. Heinssen, D. Pine, and K. Quinn. 2010. Research domain criteria (RDoC): Toward a new classification framework for research on mental disorders. *American Journal of Psychiatry* 167: 748–751. 20595427 10.1176/appi.ajp.2010.09091379

[CR38] Kendell, R. 1975. *The role of diagnosis in psychiatry*. Blackwell Scientific Publications.

[CR39] Knitzer, J. 2007. Putting knowledge into policy: Toward an infant-toddler policy agenda. *Infant Mental Health Journal* 28(2): 237–245. 28640551 10.1002/imhj.20131

[CR40] Knudsen, E., J. Heckman, J. Cameron, and J. Shonkoff. 2006. Economic, neurobiological, and behavioural perspectives on building America’s future workforce. *Proceedings of the National Academy of Sciences of the United States of America* 103(27): 10155–10162. 16801553 10.1073/pnas.0600888103 PMC1502427

[CR41] Konnoth, C. 2020. Medicalization and the new civil rights. *Stanford Law Review* 72: 1165–1267.

[CR42] Lawless, A., J. Coveney, and C. MacDougall. 2014. Infant mental health promotion and the discourse of risk. *Sociology of Health & Illness* 36(3): 416–431. 24266837 10.1111/1467-9566.12074

[CR43] Leach, P. 2017. *Transforming infant well-being: Research, policy and practice for the first 1001 critical days*. Routledge.

[CR44] Lears, T. 1983. From salvation to self-realization: Advertising and the therapeutic roots of the consumer culture, 1880–1930. In *The culture of consumption: Critical essays in American history 1880–1980*, edited by R. Fox and T. Lears, 1–38. Pantheon Books.

[CR45] Little, M. 1999. Prevention and early intervention with children in need: Definitions, principles and examples of good practice. *Children & Society* 13: 304–316.

[CR46] Maj, M. 2011. Psychiatric diagnosis: Pros and cons of prototypes vs. operational criteria. *World Psychiatry* 10: 81–82. 21633674 10.1002/j.2051-5545.2011.tb00019.x PMC3104873

[CR47] Mathis, J. 1992. Psychiatric diagnosis: A continuing controversy. *Journal of Medicine and Philosophy* 17(2): 253–261. 1588247 10.1093/jmp/17.2.253

[CR48] McAvoy, J. 2014. Psy Disciplines. In *Encyclopedia of critical psychology*, edited by T. Teo. Springer.

[CR49] Medical Board of Australia. 2020. *Good medical practice: A code of conduct for doctors in Australia*. https://www.medicalboard.gov.au/codes-guidelines-policies/code-of-conduct.aspx. Accessed 15 Sept 2025.

[CR50] Mill, J. 1982 [1859]. *On liberty*. Originally published 1859. Penguin Books.

[CR51] Mills, C. 2015. The psychiatrization of poverty: Rethinking the mental health–poverty nexus. *Social and Personality Psychology Compass* 9(5): 213–222.

[CR52] Morgan, K. 1998. Contested bodies, contested knowledges: Women, health, and the politics of medicalization. In *Agency, autonomy, and politics in women’s health*, edited by S. Sherwin. Temple University Press.

[CR53] Mulrooney, K., H. Egger, S. Wagner, and L. Knickerbocker. 2019. Diagnosis in young children: The use of the DC:0-5™ diagnostic classification of mental health and developmental disorders in infancy and early childhood. In *Clinical guide to psychiatric assessment of infants and young children*, edited by K. Frankel, J. Harrison, and W. Njoroge, 253–283. Springer International Publishing.

[CR54] Munro, E. 2011. *The Munro Review of Child Protection: Final report: A child-centred system*. Department for Education (U.K.).

[CR55] Nagle, G. 2019. Investing in early childhood development and infant mental health. In *Handbook of infant mental health*, edited by C. Zeanah. Guilford Press.

[CR56] Narayan, A., A. Lieberman, and A. Masten. 2021. Intergenerational transmission and prevention of adverse childhood experiences (ACEs). *Clinical Psychology Review* 85: 101997. 33689982 10.1016/j.cpr.2021.101997

[CR57] O’Connor, C., I. Kadianaki, K. Maunder, and F. McNicholas. 2018. How does psychiatric diagnosis affect young people’s self-concept and social identity? A systematic review and synthesis of the qualitative literature. *Social Science & Medicine* 212: 94–119. 30029092 10.1016/j.socscimed.2018.07.011

[CR58] Parens, E. 2013. On good and bad forms of medicalization. *Bioethics* 27(1): 28–35. 21535062 10.1111/j.1467-8519.2011.01885.x

[CR59] Parsons, T. 1951. Illness and the role of the physician: A sociological perspective. *American Journal of Orthopsychiatry* 21(3): 452–460. 14857123 10.1111/j.1939-0025.1951.tb00003.x

[CR60] Peters, S., R. Calam, and R. Harrington. 2005. Maternal attributions and expressed emotion as predictors of attendance at parent management training. *Journal of Child Psychology and Psychiatry* 46: 436–448. 15819652 10.1111/j.1469-7610.2004.00365.x

[CR61] Piccolo, L., and K. Noble. 2019. Poverty, early experience, and brain development. In *Handbook of infant mental health*, 4th ed., edited by C. Zeanah. Guilford Press.

[CR62] Pitts, J. 1968. Social control: The concept. In *International encyclopaedia of the social sciences*, edited by D. Sills. Macmillan.

[CR63] Purdy, L. 2001. Medicalization, medical necessity, and feminist medicine. *Bioethics* 15(3): 248–261. 11699544 10.1111/1467-8519.00235

[CR64] Radden, J. 2018. Public mental health and prevention. *Public Health Ethics* 11(2): 126–138.

[CR65] Rieff, P. 1987. *The triumph of the therapeutic: Uses of faith after Freud*. University of Chicago Press.

[CR66] Riessman, C. 1983. Women and medicalization: A new perspective. *Social Policy* 14: 3–18. 10264493

[CR67] Sadler, J., F. Jotterand, S. Lee, and S. Inrig. 2009. Can medicalization be good? Situating medicalization within bioethics. *Theoretical Medicine and Bioethics* 30: 411–425. 19997778 10.1007/s11017-009-9122-4

[CR68] Sawicki, J. 1991. Disciplining mothers: Feminism and the new reproductive technologies. In *Disciplining Foucault: Feminism, power and the body*, edited by J. Sawicki. Routledge.

[CR69] Saxena, S., E. Jané-Llopis, and C. Hosman. 2006. Prevention of mental and behavioural disorders: Implications for policy and practice. *World Psychiatry* 5(1): 5–14. 16757984 PMC1472261

[CR70] Schechter, D., E. Willheim, F. Suardi, and S. Serpa. 2019. The effects of violent experiences of infants and young children. In *Handbook of infant mental health*, 4th ed., edited by C. Zeanah. Guilford Press.

[CR71] Scott, W. 1990. PTSD in DSM-III: A case in the politics of diagnosis and disease. *Social Problems* 37(3): 294–310.

[CR72] Sholl, J. 2017. The muddle of medicalization: Pathologizing or medicalizing? *Theoretical Medicine and Bioethics* 38: 265–278. 28674861 10.1007/s11017-017-9414-z

[CR73] Sickel, A., J. Seacat, and N. Nabors. 2014. Mental health stigma update: A review of consequences. *Advances in Mental Health* 12(3): 202–215.

[CR74] Stern, D. 2000. *The interpersonal world of the infant: A view from psychoanalysis and developmental psychology*. Basic Books.

[CR75] Strong Foundations Collaboration. 2019. *The first thousand days: A case for investment*. Australian Research Alliance for Children and Youth.

[CR76] Szasz, T. S. 1960. The myth of mental illness. *American Psychologist* 15(2): 113-118.

[CR77] The Royal Australian and New Zealand College of Psychiatrists. 2023. *Building mental health and well-being in Australia and New Zealand through early support for infants, children and their families*. RANZCP.

[CR78] Thompson, S., C. Kiff, and K. McLaughlin. 2019. The neurobiology of stress and adversity in infancy. In *Handbook of infant mental health*, 4th ed., edited by C. Zeanah. Guilford Press.

[CR79] Timimi, S. 2014. No more psychiatric labels: Why formal psychiatric diagnostic systems should be abolished. *International Journal of Clinical and Health Psychology* 14(3): 208–215.

[CR80] Treichler, P. 1990. Feminism, medicine and the meaning of childbirth. In *Body/Politics: Women and the discourses of science*, edited by M. Jacobus, E. Keller, and S. Shuttleworth. Routledge.

[CR81] van Ijzendoorn, M., F. Juffer, and M. Duyvesteyn. 1995. Breaking the intergenerational cycle of insecure attachment: A review of the effects of attachment-based interventions on maternal sensitivity and infant security. *Journal of Child Psychology and Psychiatry* 36(2): 225–248. 7759588 10.1111/j.1469-7610.1995.tb01822.x

[CR82] von Klitzing, K. 2017. Should we diagnose babies? Some notes on the launch of the new zero to five classification system. *Perspectives in Infant Mental Health* 25(2): 1–3.

[CR83] Wampold, B. 2015. How important are the common factors in psychotherapy? *World Psychiatry* 14: 270–277. 26407772 10.1002/wps.20238 PMC4592639

[CR84] Wilson, H. 2002. Brain science, early intervention and “at risk” families: Implications for parents, professionals and social policy. *Social Policy & Society* 1(3): 191–202.

[CR85] Wykes, T., and F. Callard. 2010. Diagnosis, diagnosis, diagnosis: Towards DSM-5. *Journal of Mental Health* 19(4): 301–304. 20636110 10.3109/09638237.2010.494189

[CR86] Zeanah, C. 1993. *Handbook of infant mental health*. Guilford Press.

[CR87] Zeanah, C., A. Carter, J. Cohen, H. Egger, M. Gleason, M. Keren, et al. 2017. Should we diagnose babies? No! Should we diagnose disorders in babies? Yes! *Perspectives in Infant Mental Health* 25(3-4): 1–5.

[CR88] Zeanah, C., A. Carter, J. Cohen, H. Egger, M. Keren, M. Gleason, et al. 2015. “DC:0-3” to “DC:0-3R” to “DC:0-5”: A new edition. *Zero to Three* 35(3): 63–66.

[CR89] Zeanah, C., and P. Zeanah. 2009. The scope of infant mental health. In *Handbook of infant mental health*, 3rd ed., edited by C. Zeanah. Guilford Publications.

[CR90] Zero to Three. 2017. *DC:0-5: Diagnostic classification of mental health and developmental disorders of infancy and early childhood: A briefing paper*. https://www.zerotothree.org/resources/1953-dc-0-5-a-briefing-paper-on-diagnostic-classification-of-mental-health-and-developmental-disorders-of-infancy-and-early-childhood. Accessed 15 Sept 2025.

[CR91] ____. n.d. *Building strong foundations: Advancing comprehensive policies for infants, toddlers, and families*. https://www.zerotothree.org/resources/series/building-strong-foundations-advancing-comprehensive-policies-for-infants-toddlers-and-families. Accessed May 25, 2021

[CR92] Zola, I. 1972. Medicine as an institution of social control. *Sociological Review* 20(4): 487–504. 4645802 10.1111/j.1467-954x.1972.tb00220.x

[CR93] ____. 1976. Medicine as an institution of social control. *Ekistics* 41(245): 210–214.

